# Supplementary material for: In Vitro Identification of Phosphorylation Sites on TcPolβ by Protein Kinases TcCK1, TcCK2, TcAUK1, and TcPKC1 and Effect of Phorbol Ester on Activation by TcPKC of TcPolβ in Trypanosoma cruzi Epimastigotes
Source: Microorganisms. 2024 Apr 30;12(5):907. doi: 10.3390/microorganisms12050907 (PMC11124317; doi:10.3390/microorganisms12050907)
Supplement: Supplementary file 1 [file microorganisms-12-00907-s001.zip › Figure S1.pdf]

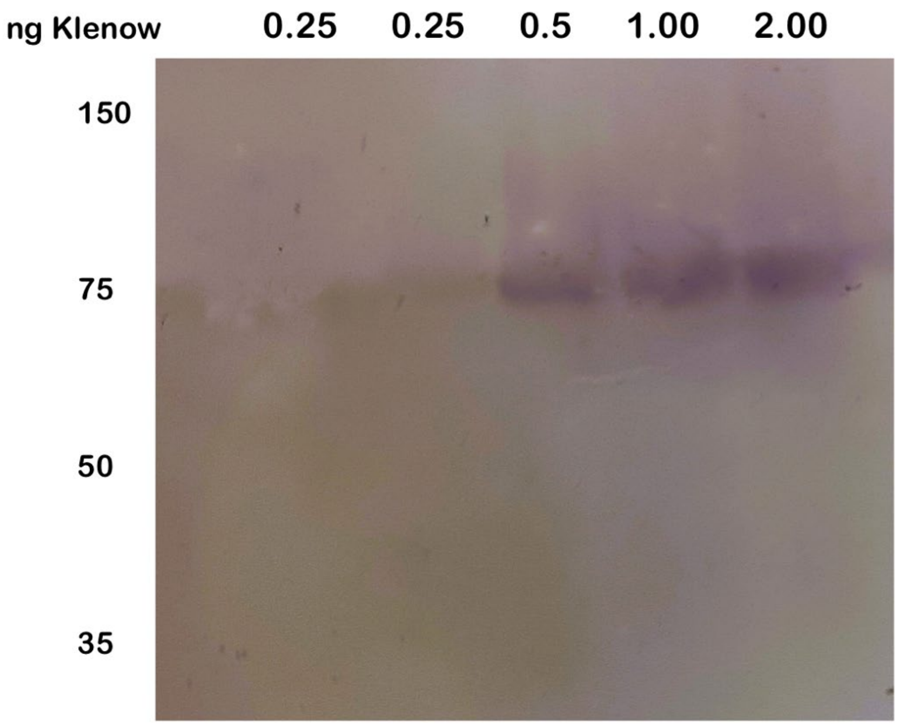

Figure S1. DNA polymerase activity gel assay of DNA polymerase Klenow large fragment. Amounts of the DNA polymerase are indicated at the top of the figure. The DNA polymerase was separated in a 10% PAGE-SDS and detected as described in materials and methods.
